# Computational tools and data integration to accelerate vaccine development: challenges, opportunities, and future directions

**DOI:** 10.3389/fimmu.2025.1502484

**Published:** 2025-03-07

**Authors:** Lindsey N. Anderson, Charles Tapley Hoyt, Jeremy D. Zucker, Andrew D. McNaughton, Jeremy R. Teuton, Klas Karis, Natasha N. Arokium-Christian, Jackson T. Warley, Zachary R. Stromberg, Benjamin M. Gyori, Neeraj Kumar

**Affiliations:** ^1^ Pacific Northwest National Laboratory (DOE), Richland, WA, United States; ^2^ Khoury College of Computer Sciences, Northeastern University, Boston, MA, United States; ^3^ Department of Bioengineering, College of Engineering, Northeastern University, Boston, MA, United States

**Keywords:** vaccine platform technologies, correlates of protection, machine learning, artificial intelligence, large language models, computational methods, data harmonization, knowledge extraction

## Abstract

The development of effective vaccines is crucial for combating current and emerging pathogens. Despite significant advances in the field of vaccine development there remain numerous challenges including the lack of standardized data reporting and curation practices, making it difficult to determine correlates of protection from experimental and clinical studies. Significant gaps in data and knowledge integration can hinder vaccine development which relies on a comprehensive understanding of the interplay between pathogens and the host immune system. In this review, we explore the current landscape of vaccine development, highlighting the computational challenges, limitations, and opportunities associated with integrating diverse data types for leveraging artificial intelligence (AI) and machine learning (ML) techniques in vaccine design. We discuss the role of natural language processing, semantic integration, and causal inference in extracting valuable insights from published literature and unstructured data sources, as well as the computational modeling of immune responses. Furthermore, we highlight specific challenges associated with uncertainty quantification in vaccine development and emphasize the importance of establishing standardized data formats and ontologies to facilitate the integration and analysis of heterogeneous data. Through data harmonization and integration, the development of safe and effective vaccines can be accelerated to improve public health outcomes. Looking to the future, we highlight the need for collaborative efforts among researchers, data scientists, and public health experts to realize the full potential of AI-assisted vaccine design and streamline the vaccine development process.

## 1 Introduction

The development of effective vaccines against pathogens is a critical priority for global health. The emergence of novel pathogens, such as SARS-CoV-2, present significant challenges to global health response strategies, highlighting the pressing need for accelerated vaccine development (1–3). Traditionally, vaccines have been developed and tested empirically by immunization with inactivated or live-attenuated microorganisms or toxins (4). While traditional approaches to vaccine development have been successful in the past, they often face challenges when dealing with rapidly evolving pathogens, especially those with high mutation rates. Traditional vaccine designs have several drawbacks including adverse reactions, safety concerns with undefined or proprietary preparations, reversion to virulence, and lengthy manufacturing timelines (5). The advent of next-generation vaccine technologies with defined antigens and delivery systems eliciting desired immune responses has revolutionized the field of vaccine design. The benefits of shifting from empiricism to rational vaccine design are already becoming apparent and offer new opportunities to address these challenges and advance our understanding of vaccine efficacy and durability (6).

State-of-the-art vaccine platform technologies, such as mRNA vaccines, viral vector-based vaccines, and structure-based antigen designs (7) have shown great potential as new vaccine candidate developments in protecting against emergent pathogens. These technologies have enabled the rapid design and production of vaccines for immediate short-term protection, as exemplified by the unprecedented speed at which COVID-19 vaccines were developed and deployed. Traditional approaches to vaccine platform selection and optimization are often time-consuming and resource-intensive, which cannot match the speed of pathogen mutation rates.

Due to the availability of exascale computing platforms, next generation hardware and advanced software infrastructure, artificial intelligence and machine learning (AI/ML), and other computational tools are becoming increasingly important in vaccine development (8–10). These computing resources and tools can be leveraged to help identify potential vaccine targets, predict vaccine effectiveness, and optimize vaccine formulations. The combinatorial problem of vaccine design for selecting antigens, platforms, adjuvants, dosage, and scheduled delivery make it challenging to test all possible parameters experimentally. AI/ML solutions to determine optimal conditions could accelerate vaccine design and development and assist in experimental refinements. For instance, ML algorithms can analyze large datasets of pathogen sequences and identify conserved epitopes that can serve as vaccine targets (11). Computational models can also simulate immune responses based on different vaccine formulations, aiding in epitope selection of promising candidates.

While we refer to several recent reviews describing the prospects of artificial intelligence (AI) and machine learning (ML) in speeding up research in vaccine design (12, 13), clinical trial design (14), and other applications of machine learning in vaccine design and development (11, 15), our review provides a comprehensive analysis of the data integration challenges and opportunities specific to vaccine development. We focus on the critical knowledge gaps in the application of next-generation vaccine technologies and computational tools and propose directions for future research to address these challenges. Importantly, He et al. highlight the importance of databases and data integration approaches supporting such AI and ML techniques (16). However, the use of ML and computational tools in vaccine development still present significant technical challenges (9, 17).

A major challenge is the aggregation of existing data and knowledge relevant for vaccine design to ensure accurate and reliable models are designed and trained using information from trustworthy resources. For example, there have been over 2,000 vaccine clinical trials registered in the U.S. alone over the past decades and there is important data scattered across different regional clinical registries globally. These data may provide insight into which factors contribute to successful vaccine design. As it is critical to understand why a vaccine was successful, systematically understanding why vaccine trials fail is pivotal for improving future research methodologies, ensuring efficient resource allocation, and enhancing public health preparedness. Lessons learned from these failures can lead to faster and more effective vaccine development in the future. This, however, requires a streamlined process for comprehensive data integration. More generally, vaccine development involves integrating data from various sources, including genomic, immunological, and clinical data, which can be heterogeneous, incomplete, or inconsistent (18, 19). Aggregating and harmonizing the contents of these resources to create a more refined and comprehensive knowledgebase is a challenging task and would require the development of novel standardized ontologies, data sharing protocols, and manual curation processes (20). Another challenge is the lack of standardized benchmarks and evaluation metrics for assessing the performance and accuracy of ML models in vaccine development (21).

**Figure 1:**
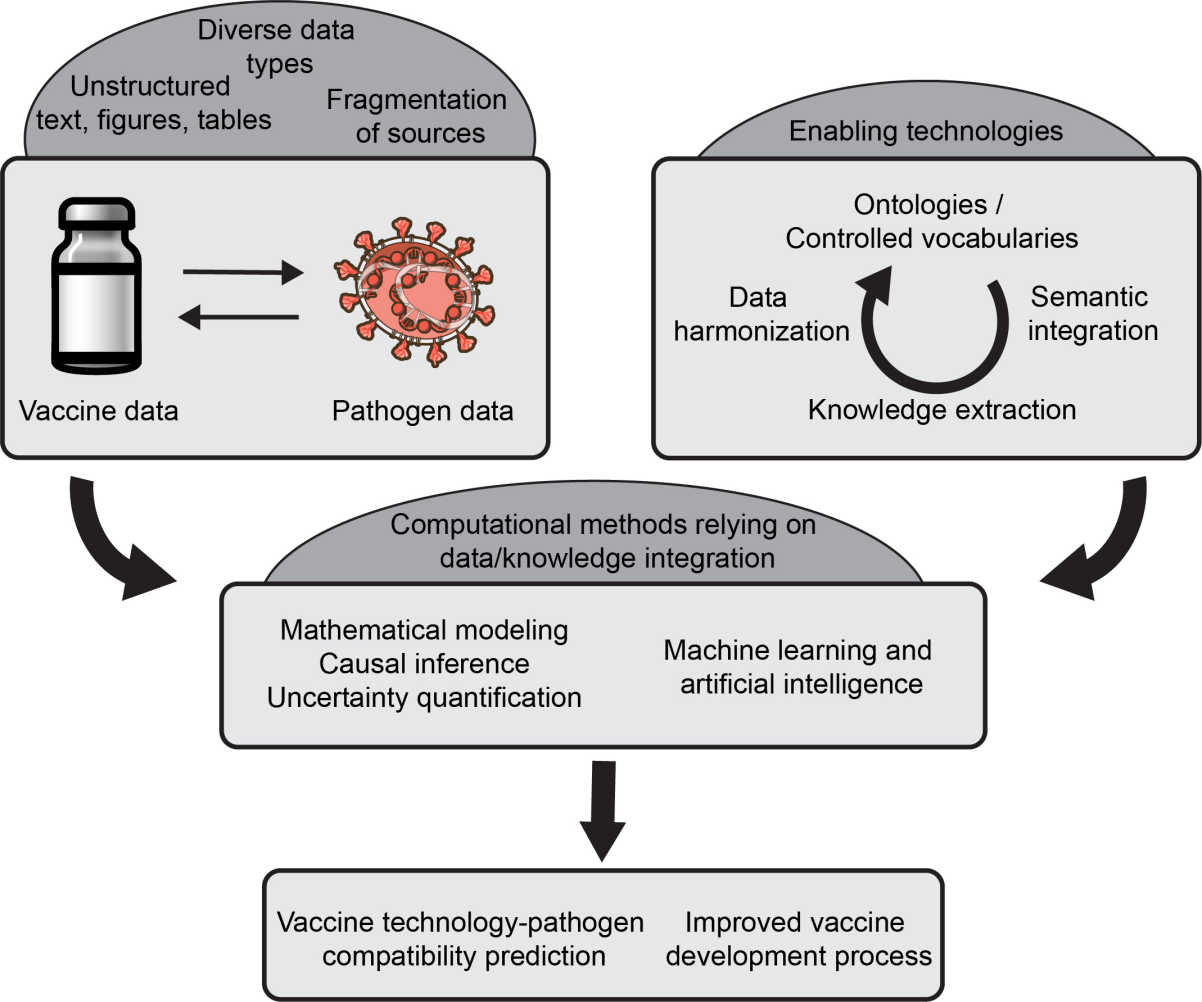
An overview of key computational methods for data integration supporting modern vaccine development. Leveraging existing data on vaccines and pathogens poses multiple challenges (top left) that can be addressed using methods for the automated extraction and integration of knowledge (top right). These enable the use of computational methods including machine learning and artificial intelligence (middle) to ultimately lead to better prediction of vaccine-pathogen compatibility and an improved vaccine development process.

Focusing on data integration, this review aims to identify and discuss critical knowledge gaps in the application of next-generation vaccine technologies and computational tools for the development of vaccines against emerging pathogens. We provide an overview of the current state of the art, highlight the challenges and limitations faced by the field, and propose directions for future research to address these challenges (**Figure 1**). Furthermore, we highlight the need for development of a novel knowledgebase that integrates diverse data sources to guide data-driven decision-making in vaccine development.

## 2 Understanding vaccine development

Emerging and re-emerging infectious diseases have threatened public health throughout history and have persisted into modern times (22). Vaccines are an important tool in the prevention of disease outbreaks, epidemics, and pandemics. In fact, the development of safe and effective vaccines against infectious diseases has been one of the most impactful scientific advances to human health of the 21^st^ century (23). However, recent climate, geodemographic, and technological shifts have altered the landscape of infectious disease risk. For example, trends in international airline travel had nearly doubled in the decade prior to the COVID-19 pandemic, increasing from two billion travelers in 2000 to over four billion travelers in 2019, leading to greater global connectivity in enabling pathogens to reach new environments and hosts (24). In addition to recent pathogenic transformation trends, there is an increased risk of infectious disease outbreaks because of delays in vaccine development and production. The vaccine lifecycle, from discovery to licensure, can cost billions of dollars and requires nearly a decade of approval processes for authorization with only an average ~6% success rate pre-pandemic (25).

**Figure 2:**
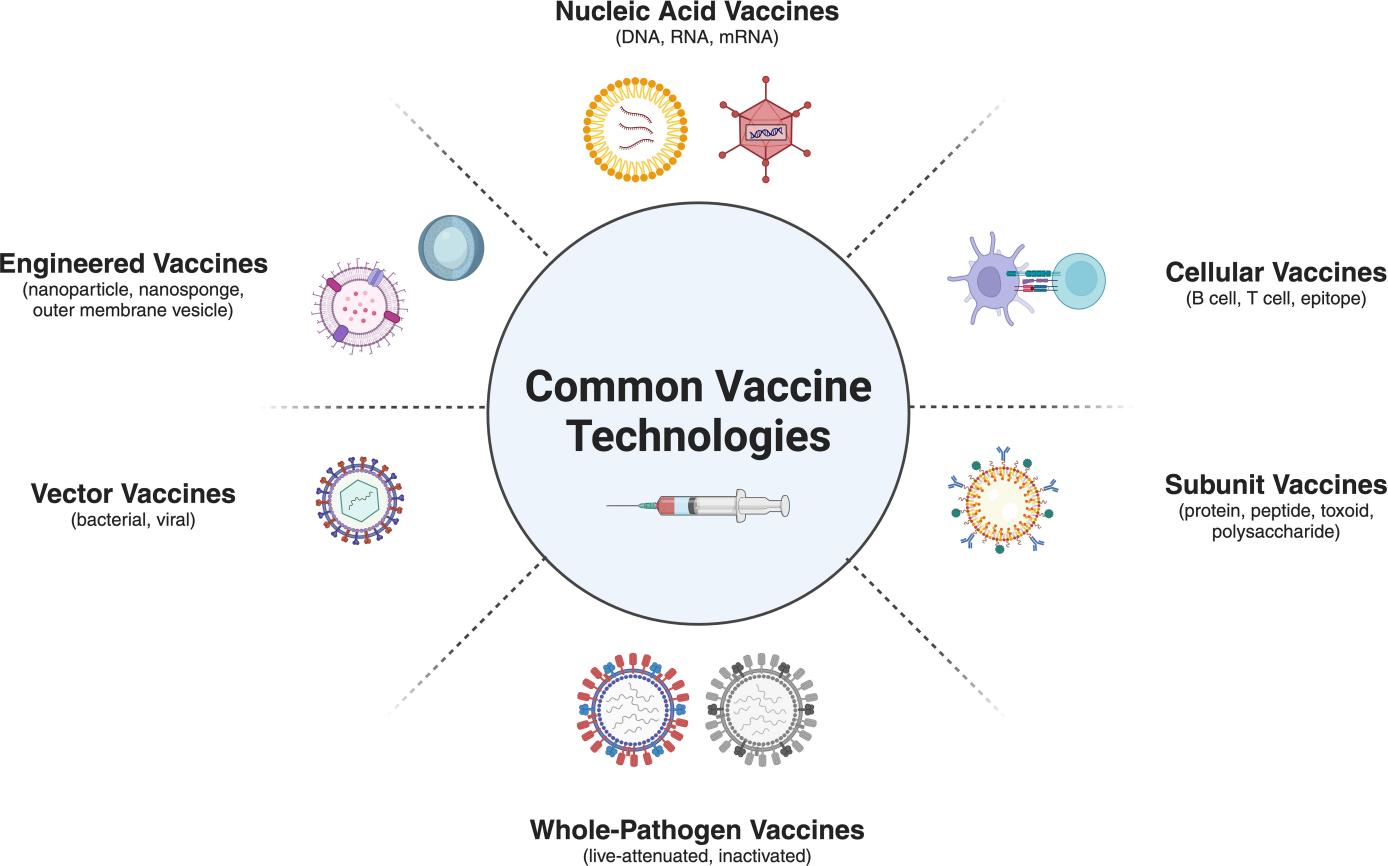
This figure showcases a variety of emerging vaccine platform technologies, including nucleic acid-based vaccines, whole pathogen vaccines, vector-based vaccines, engineered vaccine, cellular vaccines and nanoparticle-based delivery systems. The selection of an appropriate vaccine platform is a critical step in the vaccine development process and is guided by factors such as the nature of the pathogen, the desired immune response, and the target population. Each platform offers unique advantages in terms of safety, immunogenicity, and rapid manufacturing, enabling the development of targeted vaccines against a wide of pathogens. Created using BioRender.com.

A wide collection of vaccine platform technologies exists, from traditional vaccines to next-generation platforms (26, 27). Vaccine technology developments have significantly improved in eliciting targeted immune responses and have streamlined the processes enabling rapid scalability for the deployment of novel interventions. Emerging vaccine platform technologies include nucleic acid-based vaccines (28–30), recombinant vector vaccines (31, 32), whole-pathogen adapted vaccines (33–35), cellular vaccines (36), subunit vaccines (37, 38), engineered vaccines (39–41), and a suite of adjuvant-driven or synthetically derived vaccine combinations (42) leveraging the strengths of more than one platform technology (**Figure 2**). Vaccine platform technologies (43) are widely discussed but inconsistently classified as both the complexity of the technology and the scientific jargon used to describe these vaccines are disparate.

**Figure 3:**
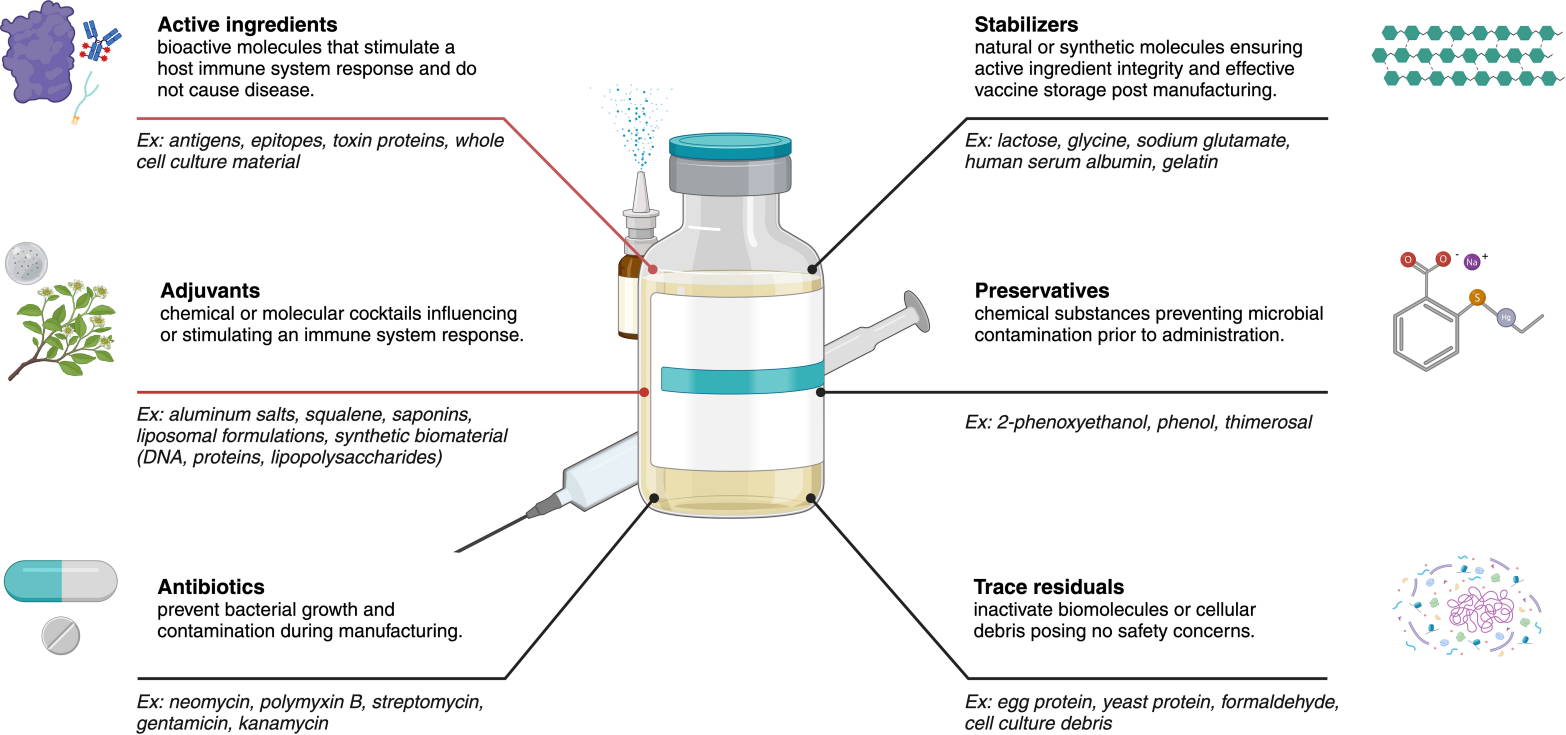
Common vaccine components (antigen, adjuvant, and delivery system) representing specific roles these ingredients play in the overall design of a given vaccine product for enhancing protection against an emerging infectious disease. Red lines indicate the two main contributors having a direct influence on vaccine efficacy and immune systems responses, where flexible combinations of these two ingredients enable improved vaccine efficacy and effectiveness depending on the optimal technology platform selected. The optimal combination of these components is crucial for developing safe and effective vaccines that elicit robust and long-lasting immune protection. Created using BioRender.com.

Vaccine platform technology selection is only one part of a more comprehensive protective design. Additional protective vaccine design components (44) have various ingredients including active substances, antibiotics, adjuvants, preservatives, stabilizers, and other trace components (**Figure 3**) (30, 45). These protective ingredients often play an immunogenic role (structural, functional, or biological) in a given design product for subsequent downstream safety and immunogenicity interrogation (46, 47).

Recent advancements in vaccine development continue to improve vaccine manufacturing and accessibility for global distribution (48). However, despite recent efforts to improve vaccine product accessibility, there remains a large gap in vaccine product information, including metadata resources required for harmonizing cross-protective vaccine study information supporting new insights from ongoing interventional studies.

The limited union of metadata standards, reporting guidelines, and shared terminologies outlined by varying governmental and scientific resources (49, 50) make it challenging to accurately capture, extract, and integrate valuable knowledge required for protective insight discovery supporting new durable vaccine designs. The rate of disseminated research results reported in journal articles, corresponding to clinical trials registered to study outcomes, is limited. This creates large gaps in quality information tracking making it challenging to identify promising preclinical vs. clinical vaccine candidate development for rapid pandemic response (51–54).

## 3 Challenges and limitations of vaccine development

### 3.1 Pathogen genetics, mutation rates, and immune evasion strategies

The early stages of vaccine development rely heavily on computational tools for pathogen surveillance, DNA/RNA sequencing, protein repertoire prediction, and epitope prediction (55–57). These tools play a crucial role in identifying potential vaccine targets and designing effective immunization strategies against emerging pathogens. Emergent lethal human viruses pose many unique challenges for vaccine design and development due to their genetic diversity, mutation rates, and immune evasion strategies (21, 58, 59). The genetic variability of emerging pathogens can impede the identification of consistent vaccine targets and can lead to reduced vaccine efficacy and the need for frequent vaccine updates. For example, RNA viruses, such as influenza and HIV, rapidly mutate, making it challenging to identify vaccine targets (60, 61). Pathogens known for their high mutation rates often have shifting antigenic properties, heavily influencing target vaccine antigen selection and efficacy. These unpredictable mutations often lead to a loss of vaccine effectiveness over time and requires new vaccine formulations. For instance, influenza virus undergoes antigenic drift and shift, necessitating annual updates to ensure protection against circulating strains (62–65).

Vaccine development efforts are further complicated depending on immune evasion strategies that pathogens employ. Pathogens evade the host immune response by antigenic variation, immune system suppression, and the shielding of vulnerable epitopes. HIV, for example, employs multiple immune evasion strategies, including the rapid mutation of its surface proteins, the masking of conserved epitopes, and the depletion of CD4+ T cells, which are critical for mounting an effective immune response (66). To overcome these challenges, vaccine development strategies should focus on identifying conserved regions of the pathogen that are less susceptible to mutation and immune evasion. This can involve the use of structure-based antigen design to create immunogens that elicit broadly neutralizing antibodies, as well as the development of novel adjuvants and delivery systems to enhance the immune response. Bioinformatics tools and machine learning algorithms can aid in the identification of potential vaccine targets and the prediction of vaccine efficacy, by speeding up the research and discovery knowledge aggregation process and eliminating manual redundancy.

### 3.2 Computational approaches to modeling and predicting correlates of protection

Correlates of protection (CoPs) are biomarkers or immune responses that are statistically associated with protection against infection or disease. The identification, estimation, and modeling of correlates of protection plays a critical role in informing the design and evaluation of vaccines and serves as a benchmark for regulatory approval and public health policy. The immune response to infection or vaccination involves complex interactions among various cell types—dendritic cells, T cells, B cells—and the molecular signals they exchange, such as cytokines and chemokines (67). Determining correlates of protection therefore presents a significant challenge in the field of vaccine development (68–71) and requires principled statistical analysis based on an understanding of the underlying immunological mechanisms. The heterogeneity of data sources and the nuances of individual immune responses pose obstacles to the reliable evaluation of CoPs, making it a specialized and challenging aspect of vaccine research. This section provides an overview of the various computational approaches to modeling and predicting CoPs, including their evaluation from clinical study results, uncertainty quantification, and data model frameworks.

Evaluating CoPs from clinical study results involves aggregating data on study designs, immunological assays, host factors, and clinical endpoints. However, the lack of standardized data formats and reporting guidelines poses challenges for integrating and analyzing this information (70, 71). Shared representational frameworks and ontologies surrounding CoPs have not yet been established, posing reproducibility challenges for data integration and statistical analyses. There is still a significant knowledge gap in identifying CoP for many vaccines, however, some CoPs have been determined for certain vaccines, such as neutralizing antibody titers for influenza vaccines (72).

CoPs enable computational analyses that support running clinical trials *in silico* and help answer a variety of relevant questions for vaccine development, such as the extrapolation of results from animal studies to humans. For example, immunobridging analysis uses correlates of protection to predict the effect of existing vaccines in protecting against a known pathogen for a given host to give insight into the effect of a candidate vaccine against a novel pathogen in a potentially different host using a different CoP (73–76). While CoPs have historically been leveraged for these analyses by *ad-hoc* methods (77–80), recent theoretical formulations of causal inference have paved the way for new generic frameworks that can be readily applied for identifying CoPs and enabling their estimation in the face of known sources of uncertainty such as unobserved confounding, sample selection bias, external validity, missing data, measurement error, variability in individual responses, and immunobridging (81–86). The next generation of methods that can provide tight bounds on the estimates of vaccine efficacy in the presence of all these sources of uncertainty (87) has the potential for high impact in the design of vaccines and clinical trials. Recent statistical methods for assessment of immune correlates of protection from randomized, controlled, vaccine efficacy trials highlighting the importance of careful experimental design planning, pre-registration, and the application of a standardized statistical analysis plans improve access to results data supporting predictive analyses (88).

#### 3.2.1 Uncertainty quantification analysis for vaccine development

Uncertainty quantification (UQ) plays a critical role in the modeling of vaccine efficacy and safety by providing a framework to assess the reliability of computational predictions. It helps in identifying the bounds within which the model’s predictions can be considered accurate, thereby guiding decision-making in vaccine development. However, quantifying uncertainty in vaccine development is challenging due to limited data, complex biological interactions, and the lack of negative data from failed vaccine candidates. In this section, we explore the role of UQ in vaccine development (89), the challenges faced, and how AI/ML tools can help in better understanding and mitigating these uncertainties.

Vaccine efficacy is typically assessed through clinical trials, where the vaccine’s ability to prevent disease or reduce its severity is evaluated. Safety is assessed through the monitoring of adverse events following immunization. Uncertainty in these models can arise from various sources, such as measurement errors, variability in individual responses, and the extrapolation of results from animal studies to humans (81). Quantifying these uncertainties helps in determining the confidence intervals around the predicted efficacy and safety outcomes, which is crucial for regulatory decision-making and risk-benefit assessments (90). Moreover, the lack of data from failed vaccine candidates in the public domain poses a significant challenge in UQ. Negative data refers to information about vaccine candidates in public domain that failed or were withdrawn at any stage of development. Understanding the reasons behind the failure of these candidates is crucial for improving future vaccine designs and optimizing resource allocation.

ML tools have emerged as powerful approaches for understanding and mitigating uncertainties in vaccine development. By integrating data from different stages of development, ML models can help identify the key factors influencing vaccine performance and provide more reliable predictions. However, it is essential to ensure that the data used for training these models is of high quality and that the models are validated using appropriate methods to avoid learning from noise. In this context, feature selection techniques can identify the most informative variables, reducing the dimensionality of the problem. By selecting relevant features, ML models can reduce the dimensionality of the problem and focus on the key factors influencing vaccine performance^1^.

Uncertainty propagation algorithms, such as Bayesian inference and Monte Carlo simulations, can quantify uncertainties associated with each component of the model (91). Surrogate modeling can approximate the behavior of vaccine models, allowing for efficient exploration of the parameter space (92). By training ML algorithms on a balanced dataset that includes both positive and negative examples, the models can learn to distinguish between successful and failed candidates more effectively (93).

#### 3.2.2 Data model frameworks for representing correlates of protection

**Figure 4:**
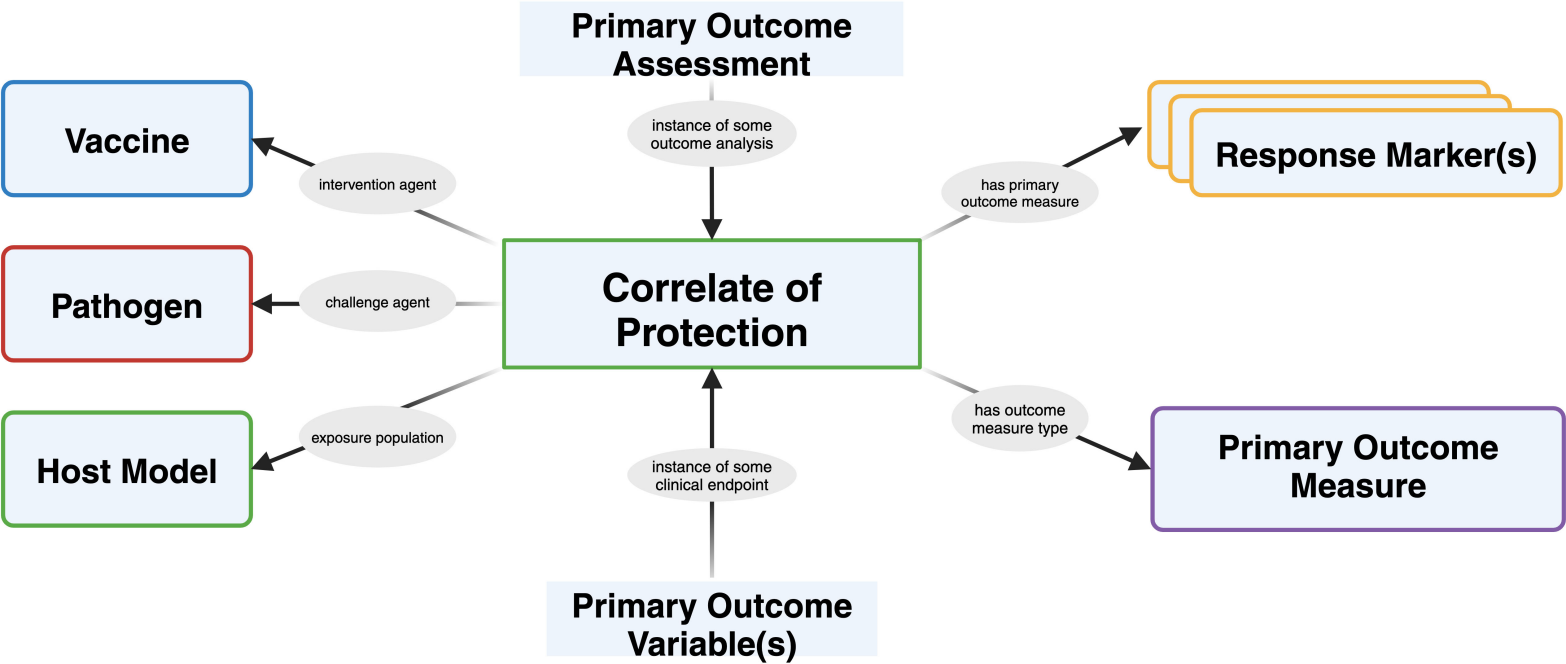
Generalized correlate of protection schema resulting from clinical trial results data and information for evaluating correlate of protection analyses. The schema integrates data from various sources such as clinical trials, including immunological assays, host factors, and clinical endpoints, to identify the key immune parameters that correlate with protection. Understanding the CoPs are essential for evaluating vaccine efficacy, optimizing vaccine design, and predicting the durability of vaccine-induced immunity. Created using BioRender.com.

The discovery of CoPs is critical for accelerating vaccine development, however, the challenges associated with their validation are significant. A multidisciplinary approach that leverages computational tools, standardized frameworks, and integrated data is required to address these challenges and advance our understanding of vaccine-induced immunity. Here, we present a prospective data model for correlates of protection outlining common data collection information elements typically required in determining correlates of protection from statistical outcomes (**Figure 4**).

Correlates of protection are semi-structured and provided mainly in literary sources (94) and reference textbooks (68), but are discussed inconsistently across sources and stem from different statistical analyses. Information reporting on immune signatures and CoPs are often spread across multiple sections of a single publication or across different publications. The data model for immune signatures (see **Figure 4**) and CoP may vary depending on the specific research questions being addressed. This lack of a universal data model makes it difficult to compare and integrate data from different studies, limiting the ability to draw comprehensive conclusions about vaccine efficacy and safety (95). Moreover, the development of advanced text mining systems that can effectively extract and integrate information from multiple sources is crucial. We discuss the challenge of scaling a common language around correlates of protection for vaccine development in the next section.

Nomenclature presents several additional challenges. For example, different types of CoPs are described using different and sometimes overlapping nomenclature such as “surrogates of protection” and “correlates of risk”, and there currently exists no taxonomical resource for CoP-related terms. Further, several different kinds of vocabularies are required to identify entities that appear as part of CoPs. The Vaccine Ontology (VO) (96) is an existing ontology that aims to represent vaccine-related information, including immune responses. However, the current VO lacks comprehensive coverage of immune response terms and relationships. Extending the VO to include a more detailed representation of immune cell types, cytokines, and signaling pathways involved in vaccine-induced immunity would greatly enhance its utility for computational modeling. Additionally, aligning immune response terms with existing ontologies, such as the Gene Ontology (GO) (97) and the Cell Ontology (CL) (98), would facilitate data integration and knowledge discovery. For example, the Infection Disease Ontology for Malaria (IDOMAL) (99) demonstrates how ontologies can be used to integrate and analyze heterogeneous data related to a specific infectious disease.

Similarly, the confluence of data and knowledge supports the development of bespoke machine learning workflows as well as the application of generic workflows. Classical machine learning and computational modeling approaches have shown initial promise in predicting vaccine effectiveness and discovering novel CoPs by leveraging immune response data (76). For example, machine learning approaches have been used to predict the immunogenicity of influenza vaccines based on the analysis of gene expression profiles and antibody titers (100).

In summary, computational approaches to modeling and predicting CoPs are essential for accelerating vaccine development and improving public health outcomes. By using clinical study results, uncertainty quantification methods, data model frameworks, and machine learning techniques, researchers can identify reliable CoPs, optimize vaccine design, and predict vaccine efficacy.

### 3.3 Data harmonization and knowledge integration challenges

**Figure 5:**
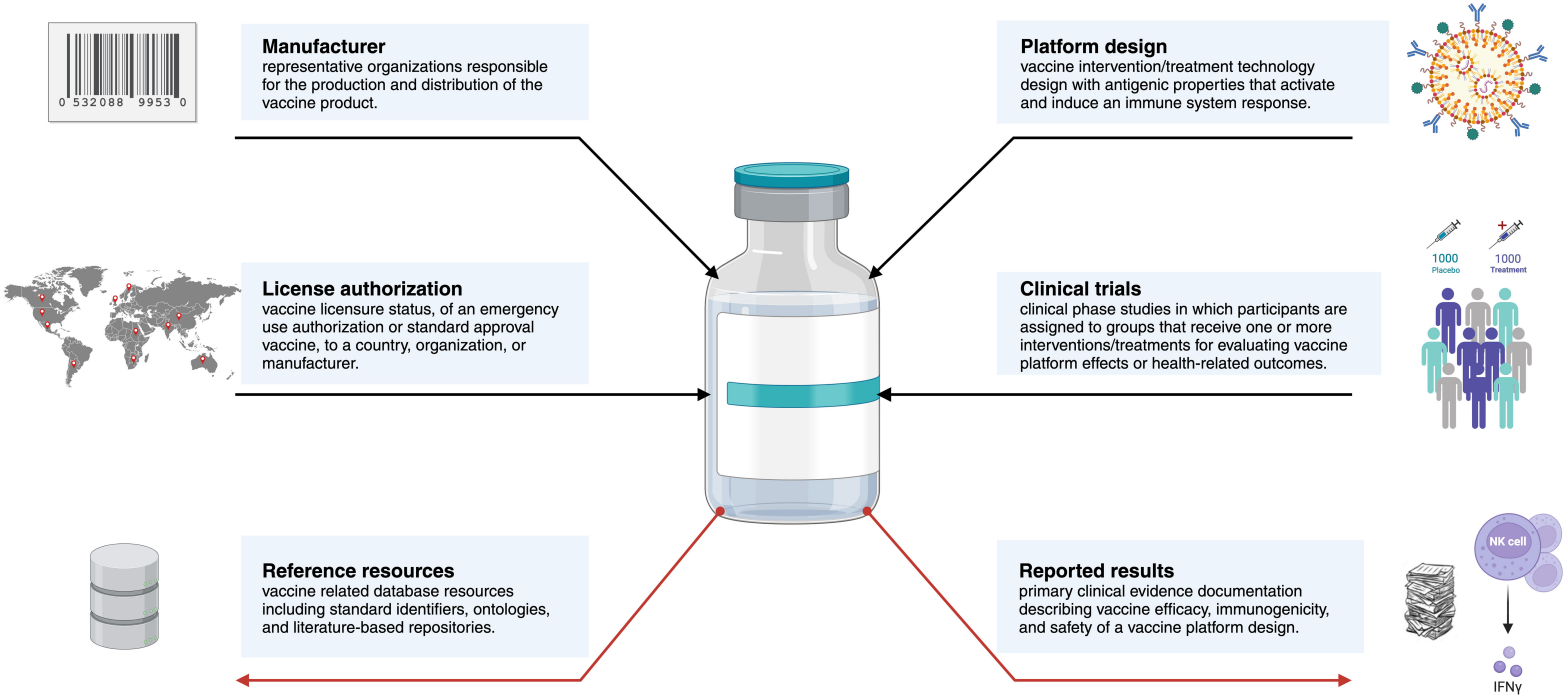
A conceptual diagram of key concepts and relationships surrounding vaccines. Red arrows indicate comprehensive methods are required for extraction and integration of these resources. Accurate curation and mapping of these vaccine products across knowledge resources is a critical step in the experimental data and knowledge harmonization for downstream query. Created using BioRender.com.

Data and relevant information describing vaccine development and immunization is distributed across dozens of distinct resources (**Figure 5**). Computational methods would generally benefit from drawing on many if not all available resources. Here we provide an overview of existing resources ranging from controlled vocabularies and ontologies to databases on vaccine characteristic and immune responses. We also point out important gaps and limitations associated with the current state of these resources.

#### 3.3.1 Resources for identifying critical vaccine relationship information

The storage and management of scientific data involves identifying concepts in an unambiguous way. Concepts relevant for vaccine development include, for instance, organisms (hosts or pathogens), vaccine products and technologies, cell types, genes, proteins, biological processes, and several other entity types. Concept identification is typically achieved by using unique identifiers that remain distinct from colloquial names and synonyms. Identifiers are assigned to concepts by controlled vocabularies, taxonomies and ontologies (101). In addition to standardizing the identification of relevant concepts, resolver resources construct links to web pages that describe each concept (102, 103). For example, the NCBI Taxonomy database (https://www.ncbi.nlm.nih.gov/taxonomy) has assigned an identification number 28450 to *Burkholderia pseudomallei*. This identifier can be resolved using the compact URI (CURIE) identifier schema standard (104, 105) as ncbitaxon:28450. Unique namespace prefixes, such as ncbitaxon assigned by the local data provider, can be persistently mapped from literature references to HTML webpage locations being described by this taxon identifier using CURIE resolver services by Identifiers.org (https://identifiers.org/taxonomy:28450) or the Bioregistry (https://bioregistry.io/ncbitaxon:28450).

Below, we summarize the landscape of such identifier and terminology resources for several concept types relevant to vaccine development.

##### 3.3.1.1 Vaccine naming and persistent identification

There exist several ontologies and related resources that catalogue and assign identifiers to vaccines. These resources provide a detailed hierarchical classification of vaccines, such as by their platform design, the pathogen against which they immunize, and the disease against which they inoculate. The most detailed of such is the Vaccine Ontology (96). Several more general resources also include vaccines such as Medial Subject Headings (MeSH) (106), the National Cancer Institute Thesaurus (NCIT) (107), the Unified Medical Language System (UMLS) (108), the Computer Retrieval of Information on Science Projects (CRISP) Thesaurus (109), Medical Dictionary for Regulatory Activities (MedDRA) (110), Logical Observation Identifiers Names and Codes (LOINC) (111), and Systematized Nomenclature of Medicine - Clinical Terms (SNOMED-CT) (112).

Additionally, several organizations maintain their own unique identification systems for vaccines, such as the United States Food and Drug Administration’s Submission Tracking Number (identifier prefix: STN), the United States Center for Disease Control and Prevention’s vaccine administered) code set (identifier prefix: CVX), the American Medical Association’s Current Procedural Terminology (identifier prefix: CPT), the European Medicines Evaluation Agency (identifier prefix: EMA) product number, and the World Health Organization Anatomical Therapeutic Chemical Classification (identifier prefix: ATC) codes (113–120). These resources are typically used to identify vaccines in reference to primary study metadata, such as clinical trials protocols and statistical plans, sponsor authorization and licensing identifiers, and product tracking numbers. Despite the existence of these resources, ensuring the persistence of identifiers and harmonized naming, standards pose a large challenge in this area (121, 122).

The broad array of agency-specific product information systems and coded identifiers make it difficult to verify and integrate identification elements across resources by country. One specific challenge is that not all data sources employ similar guidelines or best practices for assignment of resolvable identifiers and naming conventions.

##### 3.3.1.2 Vaccine ingredients and common components

The Vaccine Ontology curates terms representing components of vaccines, such as adjuvants, antigens, emulsifiers, preservatives, solvents, and stabilizers. Many of these terms are linked to chemicals in the Chemical Entities of Biological Interest (ChEBI) Ontology (123) and other related chemical identification resources. The Vaccine Adjuvant Compendium (https://vac.niaid.nih.gov) curates similar terms (124, 125), and provides additional context linking to immune signatures, pre-clinical, and clinical information. Unstructured text information exists in various *ad-hoc* formats that do not use identifiers, such as the FDA vaccine insert packets and CDC CVX code labels describing excipients. Better standardization across these sources would enable principled analysis of the role vaccine components might play in efficacy and safety.

##### 3.3.1.3 Vaccine host-pathogen taxonomy

The NCBI Taxonomy repository provides a comprehensive and detailed hierarchical classification of organism-specific lineages across all clades of life (126). It is particularly useful for annotating host and pathogen organisms as well as vaccine targeted organisms based on genomic sequence identification. However, genomic database collections do not always track and assign persistent identifiers to pathogenic variants of interest. This can create critical gaps for assembling vaccine-pathogen relationships connected to variant-specific sequence identities during outbreaks and pandemics (127).

Variant tracking, depending on the mutation rate of the pathogen of interest, often requires full descriptive reporting standards when publishing experiments or observations for correctly identifying variant protection. New ontologies, such as the Coronavirus Infectious Disease Ontology (CIDO) (128), have begun to incorporate agency-specific terminology for well-known SARS-CoV-2 pathogenic variants. For example, CIDO has included new terms for common SARS-CoV-2 variant names based on the GISAID (129, 130), PANGO (131–133), and WHO classification systems (134), facilitating keeping track of circulating pathogen lineage metadata information. Accurate and up-to-date tracking of emerging pathogen variants of interest (VOI) and variants of concern (VOC) is crucial for future vaccine development success and characterization of linked protective outcomes such as vaccine efficacy and protective durability (135).

Other relevant ontologies include the Infectious Disease Ontology (IDO) collection which is connected to widely used more general disease ontologies (136). Though, currently incomplete, IDO has established a roadmap for providing curated terms for resolving pathogenic strain information in the future.

##### 3.3.1.4 Vaccine antigen selection

Vaccines can contain portions of nucleotide or peptide sequences bearing a variety of roles such as being an antigen, conjugate, or vector. Biologically-relevant genomic sequence variance and antigen coded sequence mutations are known to heavily influence vaccine design efficacy (28, 137). Explicit vaccine sequence information is valuable for downstream analysis but is also often not defined nor referenced explicitly from publications, clinical trials, or other documents describing experimental approaches to new vaccine development. Antigens often correspond to a well-defined gene sequence, either identified through gene nomenclature resources such as Entrez Gene Database, protein nomenclature resources such as UniProt, or when available complete genome reference databases such as GenBank and related sequence-based database extensions (138–141). Other lookup resources specific to antigen search and discovery include the Immune Epitope Database and the VDJdb curated database of T-cell receptors with known antigen specificity (142, 143).

##### 3.3.1.5 Vaccine adverse events and discourse

Structured information on vaccine adverse events is key to assessing vaccine safety. Several related resources curate identifiers and names for adverse events, such as the Ontology of Adverse Events (OAE), Ontology for Vaccine Adverse Events (OVAE), Common Terminology Criteria for Adverse Events (CTCAE), and the Adverse Outcome Pathway (AOP) framework (144–147). Similarly, the Symptom Ontology and the Human Phenotype (HP) Ontology cover partially overlapping concepts that appear in adverse event resources and can be linked in many cases (148–150). Finally, there are resources dedicated to standardizing public discourse on vaccine effects such as the Vaccine Misinformation Ontology (VAXMO) (151).

It is important to note that extracting useful information from social media posts about vaccines is challenging due to the prevalence of misinformation and disinformation. Distinguishing genuine adverse event reports from false or misleading information remains a significant hurdle, even with the inclusion of image evidence or the use of advanced AI systems. Researchers and public health officials must exercise caution when relying on social media data for vaccine safety monitoring and develop robust methods to validate the information obtained.

##### 3.3.1.6 Vaccine-related ontology integration and gaps

The proliferation and heterogeneity of vaccine resources presents several challenges during data integration. We have given an overview of a number of resources relevant to vaccine data, still, multiple further ontologies can be found in databases that catalogue biomedical identifier resources including the Bioregistry and BioPortal (152, 153). The usage and integration of these resources, however, remains challenging. First, retrieval presents a major issue as resources appear in a variety of formats. For example, most organization-specific identification systems require web-scraping and are difficult to process. Further, several resources have licenses that are restrictive in terms of usage allowed (e.g., UMLS) or are proprietary (e.g., CPT) which makes their reuse difficult (154). Second, reconciliation is challenging, as nomenclature is not always consistent nor detailed enough to resolve ambiguities. Precise and comprehensive semantic mappings are required to reconcile ontologies and related resources at scale, which also poses several problems with respect to the availability and completeness of mappings as well as the methods necessary to accomplish this. Finally, completeness is an important issue, as even the combination of all resources does not cover all vaccines, vaccine candidates, and vaccine platforms. This can be addressed in some cases by contributing or suggesting to the maintainers of the resource to include new terms. For example, the Vaccine Ontology is a part of the Open Biological and Biomedical Ontologies (OBO) Foundry, a set of community-maintained ontologies with shared curation and community guidelines (155). In other cases, it may be required to develop new ontologies or nomenclature resources.

#### 3.3.2 Data integration methods to support machine learning

Modern life science knowledge discovery requires the integration of data and knowledge from heterogeneous, multi-modal data sources (156, 157). Data integration is becoming increasingly important to support AI/ML which leverage such heterogeneous, multi-modal data. Integration, however, is limited by variations in both structured and unstructured data formats where common data models and standards would be needed (158–160). In this section, we review current methods and best practices for data integration, highlight how they have been applied in vaccinology, and highlight upcoming challenges and opportunities for the domain. Specifically, we cover existing standards, the landscape of existing knowledge sources, and opportunities for applying natural language processing (NLP) and large language models.

Knowledge, often in the form of relationships between entities described in the previous section, are often scattered across many structured, semi-structured, and unstructured information resources. Fragmentation of structured data contained in specific vaccine related knowledgebases such as the U.S Centers for Disease Control and Prevention (CDC), Federal Drug Administration (FDA), WHO International Clinical Trials Registry Platform (ICTRP), ClinicalTrials.gov, Vaccine Adverse Event Reporting System (VAERS), etc. (113, 115, 161, 162), along with reported outcomes sparingly shared in resulting journal articles, are a challenge to track down and connect outcome and study phase progress. More specifically, there are a variety of disparate data formats and standards across published experimental studies on pathogens and vaccine platforms. Structured biomedical data and clinical study knowledge can appear in several formats. Simple relationships between concepts can be encoded as semantic triples consisting of a subject, a predicate and an object, possibly further augmented by additional properties (163). Representing complex knowledge such as correlates of protection requires a more detailed data model for accurately capturing the necessary biological entities and the relationships they represent.

A handful of databases contain host-pathogen data aggregated from literature in a structured form. Efforts from the Human Immunology Project Consortium (HIPC) database (https://immunespace.org/), the Host-Pathogen Interaction Prediction (HPIP) analysis framework, and the COVID-19 Prevention Network (CoVPN) consortium network website (https://preventcovid.org/) (164–167) collect and curate relevant information, but there is currently no comprehensive resource containing detailed immune signature identifications and CoP statistical results.

The NIH 2023 data sharing policy (https://grants.nih.gov/grants/guide/notice-files/NOT-OD-21-013.html) is expected to have a significant impact on the availability of raw data in public repositories. This increased access to data will facilitate the development and validation of computational tools and data integration methods for vaccine research. The policy mandates that all NIH-funded research generate publicly accessible data, which will enable researchers to more easily combine and analyze data from multiple sources. This, in turn, will accelerate the identification of novel vaccine targets, the optimization of vaccine formulations, and the assessment of vaccine safety and efficacy.

##### 3.3.2.1 Host-pathogen infection, disease, and clinical outcome data

Relationships of vaccines to pathogens, host organisms, and diseases constitute the backbone of vaccine-related knowledge. However, this information is highly fragmented, sparse, and available at varying levels of granularity. For example, connections between vaccines and the organisms they immunize against can be found in a combination of structured sources like VO, VIOLIN (168), and the Cov19VaxKB (169) combined with unstructured sources published by organizations like the CDC, FDA, and EMA. The VO and VIOLIN database provide explicit annotations on vaccines’ host organism(s), which are typically implicit in other resources. Connections between vaccines and diseases can also be extracted from a combination of direct structured annotations in VO and VIOLIN, through inference on clinical trial resources, inferred through the Disease Ontology’s *has material basis in* annotations, and from unstructured sources from the CDC, FDA, and EMA.

Vaccine side effects and adverse outcomes are available from multiple resources. VAERS accumulates adverse event reports, which requires statistical interpretation and only covers a small number of vaccines. Further, it does not use controlled vocabularies and therefore needs preprocessing (grounding). Additional processing has been done to match VAERS to the Adverse Event Ontology (AEO) (170). VAERS has also been analyzed with other natural language processing systems for text classification to medical officer review (171). VIOLIN contains side effect information in unstructured text that can be extracted with NLP. Side effects can also be extracted from the FDA’s label inserts for vaccines (172). NLP has been shown to be an effective method approach for vaccine event extraction (173, 174), while ML methods have been shown to be an effective approach for side effect prediction-based methods using electronic health records (175).

Despite the availability of these sources and inference methods, it’s necessary to do additional manual curation to achieve full coverage of the vaccine landscape. Methods that measure entity co-occurrence, such as those based on NLP, could provide an initial assessment of the landscape accelerating manual curation.

##### 3.3.2.2 Clinical trials data

Clinical trials data is distributed across a large number of country- and region-specific registries (176). The principal clinical trial registry used in the United States, ClinicalTrials.gov, contains the most granular information on study results (177). The World Health Organization (WHO) aggregates multiple clinical trial registries into a unified data store and provides cross-references when the same trial is registered in multiple primary registries (178). However, there exist several challenges in using the collection of these registries due to differing data availability, data standards, and curation practices used in each of the constituent clinical trial registries. For example, some registries contain a dedicated field for the phase of a clinical trial, some contain it within free text describing the trial, and some do not include it at all. Further, to connect information between trials and other resources, concepts need to be standardized to controlled vocabularies or ontologies. ClinicalTrials.gov uses MeSH to accomplish this for its trials’ conditions and interventions, but there remain significant gaps in standardizing these and other fields.

Examples of successful data harmonization efforts and their benefits more generally include population, intervention, comparison, and outcome (PICO) framework (179). The development and adaptation of external data standards such as Clinical Data Interchange Standards Consortium (CDISC) (https://www.cdisc.org) and BRIDG (https://bridgmodel.nci.nih.gov) is one avenue towards standardizing data before it enters registries. Alternatively, several complementary approaches have demonstrated progress towards establishing data models and information extraction pipelines covering several aspects of clinical trial registries, including for outcomes (180), for funding sources (181), for endpoints (179), and for related regulatory documentation (182). Similar efforts to support precision medicine have resulted in clinically-enriched knowledge graphs (183, 184). Because many fields within clinical trial registries are stored as free text, there remain several opportunities for developing further data models and extraction pipeline for additional aspects, such as cohort recruitment and exclusion, intervention administration, reasons for stopping trials, and other fields. 

##### 3.3.2.3 Vaccine licensing data

Vaccine regulatory information is crucial for informing the development of new vaccines. However, the vaccine regulatory landscape is complex due to the process of approval (e.g., standard vs. emergency use authorization), the variety of license statuses (active, inactive, withdrawn, etc.), and the number of regional- and country-specific agencies that review and grant authorization. These complexities create challenges in data harmonization efforts across license tracking statuses and reporting resources.

The FDA includes detailed documentation about the review of each vaccine including its clinical review memo, approval letters, and other supporting documents. For example, the approved BioNTech COVID-19 vaccine COMIRNATY is described by the FDA in https://www.fda.gov/vaccines-blood-biologics/comirnaty (185). Similarly, the emergency use authorized Novavax vaccine is described by the FDA in https://www.fda.gov/vaccines-blood-biologics/coronavirus-covid-19-cber-regulated-biologics/novavax-covid-19-vaccine-adjuvanted (186). Despite providing detailed vaccine insert documentation for individual vaccines, the FDA does not provide a single source for aggregating versioned documentation of current approvals, emergency use authorizations, or withdrawals in a stable user-friendly location. The CDC’s CVX code resource provides an aggregated overview of *active*, *inactive*, and *never active* vaccines in the USA, but does not link to FDA approval identifiers for harmonizing release versioning. The EMA provides a single aggregated document on all reviewed medicines, including vaccines, but varies in the language used to describe these statuses.

Additionally, several third-party resources exist that aggregate or curate vaccine licensing information. For example, the Vaccine Ontology contains annotations for USA-licensed vaccines. VIOLIN stores licenses in a semi-structured way. Finally, the COVID-19 Vaccine Tracker (https://covid19.trackvaccines.org) contains detailed licensing information for SARS-CoV-2 vaccines, covering the entire complexity of the licensing landscape, but it is limited by pathogen, requires web scraping and data standardization, and its maintenance has been discontinued as of December 2022.

There remain several ongoing challenges in leveraging vaccine licensing information. First, there is the limited availability of vaccine information by distributor, which is currently scattered across unstructured sources and agencies in many regions, stored in proprietary resources inaccessible to the general research community. Second, is the variability and inconsistency of the terminology used in referencing, naming, or describing of vaccine information. Controlled vocabularies, such as the NCI Thesaurus (NCIT) collection (http://purl.obolibrary.org/obo/ncit.owl) have an incomplete classification of licensing that could serve as a basis for extension to a vocabulary that could help standardize across agencies and regions (see https://bioregistry.io/ncit:C118405). Further, common terms such as emergency use authorization (EUA) have different context- and agency-dependent meaning. Finally, capturing license information is confounded by the dynamical nature of licenses which can change over time, motivating the development of a more sophisticated data model for capturing the lifecycle of a given vaccine.

##### 3.3.2.4 Vaccine platform complexity

Vaccine design is a complex process that involves the selection of appropriate platforms, adjuvants, and antigens. Vaccine platforms are the backbone of vaccine development, providing the foundation for the delivery of antigens and the stimulation of the immune system. However, the complexity and diversity of vaccine platforms make ontologizing a challenging task. Many vaccines build on aspects of multiple platforms, and the lack of standardized ways to annotate these platforms hinders data integration and analysis. Adjuvants are essential components of many vaccines, enhancing the immune response and improving vaccine efficacy (47, 187). Antigens are the key components of vaccines that trigger the immune response and confer protection against pathogens. The lack of standardized ontologies and annotation systems for vaccine platforms, adjuvants, and antigens presents significant challenges in vaccine design and data integration. Collaborative efforts to develop and implement standardized ontologies, along with the integration of vaccine data from multiple sources, are essential for advancing vaccine research and development. By leveraging the power of ontologies, unique and persistent identifiers, with results data for integration we can accelerate the design of safe and effective vaccines and improve public health outcomes.

#### 3.3.3 Vaccine platform data curation knowledge gaps

As discussed above, significant knowledge gaps exist in the curation of data on vaccine platforms, stemming from language and reporting inconsistencies, the lack of standardized datasets, and variable data reporting requirements. These gaps hinder the ability to rapidly develop and adapt vaccines to new pathogens, as exemplified by the challenges faced during the COVID-19 pandemic. The use of human-readable formats, such as unstructured text in scientific articles, makes it difficult to extract and integrate data across different studies. Additionally, the inconsistent use of terminology between experimental and computational domains creates barriers to data harmonization and analysis (188). Publicly available datasets on vaccine efficacy and safety often suffer from incompleteness and lack of standardization. Relevant information, such as adjuvant formulations and dosing schedules, is often reported in an *ad-hoc* manner and scattered across different sources, including clinical trial registries, journal articles, and regulatory documents (189).

Efforts to promote the use of machine-readable formats, such as standardized data tables and structured metadata, can improve the efficiency and accuracy of data curation. The development of tools and platforms for automated data extraction and integration, leveraging natural language processing and machine learning techniques, can help overcome the challenges posed by unstructured and heterogeneous data sources.

#### 3.3.4 Community standards, data sharing, and reporting

There exists a confluence of community data standards, model formats, and reporting guidelines that support the curation and organization of knowledge from primary sources (190). Many such curated resources are adopting external data standards to improve their reusability. For example, the molecular interaction and pathway modeling community have several standard data exchange language formats for encoding curated artifacts, including BEL, BioPAX, and SBML standards (191–193). The clinical data modeling community has also produced several standards for encoding clinical data, including the CDISC, the Observational Medical Outcomes Partnership Common Data Model (OMOP-CDM), and the HL7/FHIR (194–196).

Concurrently, several general principles, guidelines, and community standards resources have emerged to support curators (197). Popular implementations include the FAIR (findable, accessible, interoperable, reusable) data principles, TRUST (transparency, responsibility, user focus, sustainability and technology) principles, CARE principles (CARE Principles for Indigenous Data Governance), FAIR for Research Software (FAIR4RS), O3 (open data, open code, open infrastructure) guidelines, the Research Data Alliance COVID-19 working Group (RDA COVID-19) standards collection, and structured data extaction APIs such as Google Colab (https://ai.google.dev/gemini-api/tutorials/extract_structured_data) (198–203).

Frameworks that automate the assembly of biomedical knowledge can support building new databases and knowledge graphs that can be queried from a combination of programing languages, dialog systems, and more recently, through the interface of large language models. This includes schemas such as the Biolink Model, BioCypher, Phenotype Knowledge Translator (PheKnowLator), GA4GH Phenopackets, and ISA-FHIR (204–208). Other approaches towards knowledge assembly based on the semantic web (209, 210) and linked open data (211, 212) allow for information to be federated from many distinct sources following shared data modeling practices. A well-known example is the UniProt RDF platform and SIB linked data (213–215).

### 3.4 Automated tools for knowledge extraction from literature

A key challenge of data integration in this space (and life sciences more broadly) is that most data is fragmented across scientific publications in an unstructured form such as text, figures, and tables. Peer-reviewed publications relevant to vaccine mechanisms are accessible via PubMed and PubMed Central, as well as publisher-specific repositories, however, most content is not available in a full-text form. Some targeted projects such as LitCovid provide SARS-CoV-specific publications but are limited to only a subset of all relevant literature (216). Given access to literature content, natural language processing techniques can extract data from text in a structured form. Natural language processing systems typically approach extraction from scientific publications by first recognizing key concepts in text, a process called named entity recognition (NER), then extracting relationships between concepts (called relation extraction) (217). Traditionally, NLP systems have used rule-based extraction approaches whereby patterns corresponding to relations of interest are matched to trigger extraction logic from text (218). More recently, machine learned, transformer-based algorithms have become prevalent. These models (including PubmedBERT (219) and BioBERT (220)) are generic to processing biomedical text and can be fine-tuned for specific extraction tasks. Finally, the latest generation of large language models – both proprietary (e.g., ChatGPT, https://chatgpt.com) and open source (e.g., Bloom (221)) – can be used for interpreting and extracting data from publications without fine tuning, rather, using custom prompts with instructions and examples, sometimes called in-context learning (222). We highlight prior work on NLP applied in the domain of vaccine mechanisms in **Table 1**.

**Table 1:** Prior applications of natural language processing in the domain of vaccine mechanisms.

Natural Language Processing Task	Reference(s)
Recognizing adverse events	(171, 223–228)
Processing social media content on vaccine response and sentiment	(229–234)
Identifying immune signatures and underlying biological processes	(235–238)
Identification and prioritization of relevant literature	(239–241)
Processing of clinical trial registries	(242)
Identifying relevant dataset sequence links	(243)
Topic clustering and analysis	(244, 245)
Identification of named entities	(246, 247)
Constructing knowledge graphs	(248, 249)
Human-machine health interactions	(250)
PICO extraction	(251)

Recent advancements in LLMs, such as ChatGPT, have shown promising results in various scientific domains, including single-cell RNA-seq analysis (252). In addition to using LLMs for data extraction tasks, as outlined above, LLMs may be used directly as a source of integrated vaccine knowledge. However, due to the nature of LLMs capturing relationships in text implicitly through learned weights, it is challenging to connect responses generated by LLMs to primary evidence from literature or other sources. Therefore, further investigation is needed to assess the extent to which these models can provide accurate and reliable information in this context.

Correctly identifying and extracting data information from semi-structured tables, graphs, and figures has proven to be challenging for NLP and other automated approaches. NLP methods present generic- and domain-specific challenges. Some are a result of the complexity of language itself, for example, anaphora (references to previously mentioned concepts via pronouns) and synonyms both impact the ability for rule-based systems to recognize concepts correctly. Similarly, imprecise language (e.g., varying specificity of vaccine terms) and ambiguity (e.g., “NLP vaccine” nomenclature) are difficult for even newer systems to overcome. As LLMs and other machine-learned techniques become more prevalent, the need for reproducible and reliable workflows becomes important as the same inputs are no longer guaranteed to give the same outputs, and the issue of hallucination can affect the accuracy of results. Hallucination, in the context of LLMs refers to the generation of false or misleading information that is not grounded in the input data or the model’s training. This can lead to inaccurate or unreliable outputs, which is a significant concern when applying these models to scientific research. Finally, a practical issue for all NLP methodologies is the limited availability of full-text content since important data or tables often do not appear in the abstract (253).

We identify several opportunities for the application of NLP in the future. First, it can be used to generate more detailed indexes of vaccines and other concepts of interest in the literature to make it easier to query, enable preliminary analyses such as the assessment of relative entropy (254), and provides a foundation for further extraction and curation activities. Accordingly, there is an opportunity for using NLP to assist in automated or semi-automated relation extraction, for example, between vaccines and their target pathogens, diseases, and other features mentioned above. This presents an opportunity both in the biomedical literature as well as semi-structured datasets that contain free text fields such as those appearing in clinical trial registries and adverse event databases. Finally, we see an opportunity to use LLM-based workflows such as SPIRES (255) or Kor (256).

## 4 Conclusion

The development of safe and effective vaccines is a critical public health priority, and the integration of diverse data and knowledge and the application of AI and ML techniques hold immense promise for accelerating this process. The process of developing safe and effective vaccines is fraught with challenges, ranging from the rapid mutation of pathogens to the lack of standardized data integration and curation practices. By leveraging data from various sources, including platform data, pathogen data, and published knowledge, we can gain a more comprehensive understanding of the factors that influence vaccine efficacy and safety as shown in **Figure 1**.

This review highlights the current landscape of vaccine development, and the opportunities associated with integrating diverse data types to enable AI and ML techniques. We have discussed the role of semantic integration, causal inference and natural language processing, in extracting valuable insights from published literature and unstructured data sources. Furthermore, we have emphasized the importance of establishing standardized data formats and ontologies to facilitate seamless integration and analysis of heterogeneous data. However, to fully harness the potential of AI/ML and computational tools in vaccine development, it is crucial to address the lack of data and knowledge interoperability across various domains. This lack of integration hinders the development of comprehensive models and limits the ability to derive meaningful insights from the available data. To overcome the challenges associated with data and knowledge integration in vaccine development, we propose the following future directions and implementation strategies:

- Establishing standardized ontologies/data formats. Developing standardized ontologies and data formats specific to vaccine development can facilitate the integration of data from various sources, such as host-pathogen interactions, clinical trials, and vaccine design. This will enable more efficient data sharing and analysis across different research institutions.
- Promoting data sharing and collaboration. Encouraging a culture of data sharing and collaboration among researchers, industry partners, and public health organizations can help break down silos and facilitate the integration of knowledge across different domains. This can be achieved through the creation of open-access databases, data-sharing platforms, and collaborative research networks.
- Advancing AI/ML algorithms for data integration. Investing in the development of advanced AI/ML algorithms specifically designed for integrating heterogeneous data from disparate sources can help overcome the challenges associated with data harmonization. These algorithms should be able to handle the complexity and variability of vaccine and platform related data and provide meaningful insights to guide vaccine development.
- Integrating real-world evidence. Integrating real-world evidence, such as post-marketing surveillance data and electronic health records, with traditional vaccine development data can provide a more comprehensive understanding of vaccine safety and effectiveness. Such efforts require the development of robust data infrastructure and the application of AI/ML techniques to analyze and derive insights from these diverse data sources.
- Fostering interdisciplinary collaboration. Encouraging collaboration among experts from various fields, such as immunology, virology, data science, and computer science, can help bridge the gaps in knowledge and facilitate the development of innovative solutions to address the challenges in vaccine development.

In conclusion, realizing the full potential of AI/ML and computational tools in vaccine development will require a concerted effort from all stakeholders, including researchers, funding agencies, industry partners, government organizations, and academic institutions. By investing in the development of standardized ontologies and data formats, promoting data sharing and collaboration, advancing AI/ML algorithms for data integration, integrating real-world evidence, and fostering interdisciplinary collaboration, we can accelerate the development of safe and effective vaccines and improve global public health outcomes.
